# Supplementary material for: Fourier ring correlation simplifies image restoration in fluorescence microscopy
Source: Nat Commun. 2019 Jul 15;10:3103. doi: 10.1038/s41467-019-11024-z (PMC6629685; doi:10.1038/s41467-019-11024-z)
Supplement: Supplementary file 1 — Supplementary Information [file 41467_2019_11024_MOESM1_ESM.pdf]

SUPPLEMENTARY INFORMATION: FOURIER RING CORRELATION SIMPLIFIES IMAGE RESTORATION IN FLUORESCENCE MICROSCOPY

KOHO ET AL.

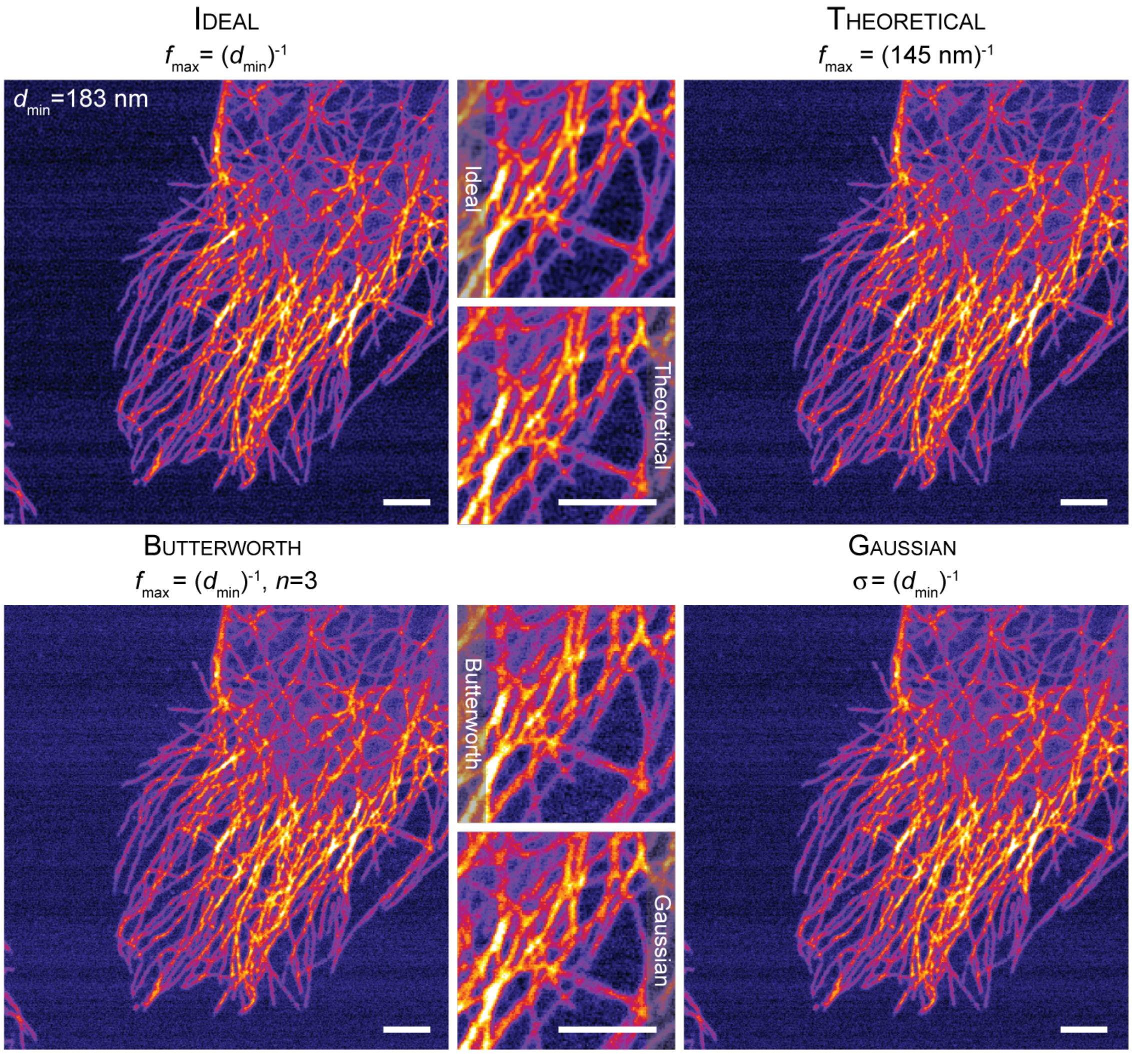

Supplementary Figure 1. *Different frequency domain low-pass filters are compared.* An image of a fixed HeLa cell is filtered with three different low-pass filters: Ideal, Butterworth and Gaussian. For the Theoretical image the Ideal filter was used, but instead of FRC, the cut-off was calculated from the microscope and sample parameters, taking into account that the image was acquired with a closed pinhole (0.4 AU) confocal ($d_{min} = 0.4\lambda/\mathrm{NA}$; $\lambda = (488\ \mathrm{nm} + 530\ \mathrm{nm})/2$; NA $= 1.4$). Scale bars 3 μm.

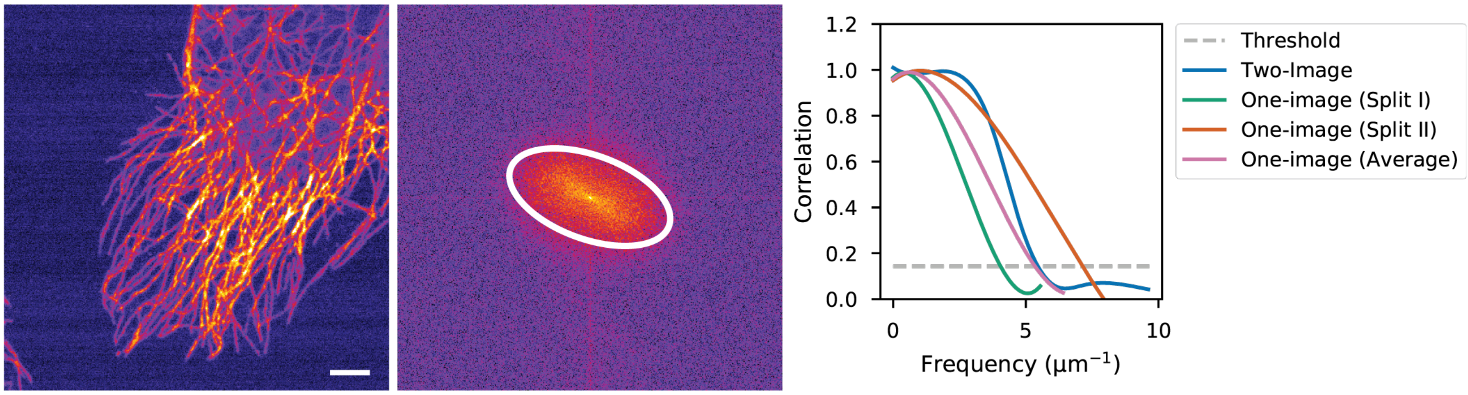

Supplementary Figure 2. *Regarding FRC and special symmetries.* The one-image FRC measures are averaged for two 90 degrees rotated splitting patterns, to compensate for possible frequency domain asymmetries, such such as the microtubulin stained HeLa cell image here. The features in the image are mainly oriented to a certain direction, which produces an oval shaped power spectrum. This asymmetry is evident in one-image FRC measures of the two sub-image pairs (Split I and Split II). Averaging the two measures is shown to match one-image FRC with the two-image FRC. Scale bar 3 μm.

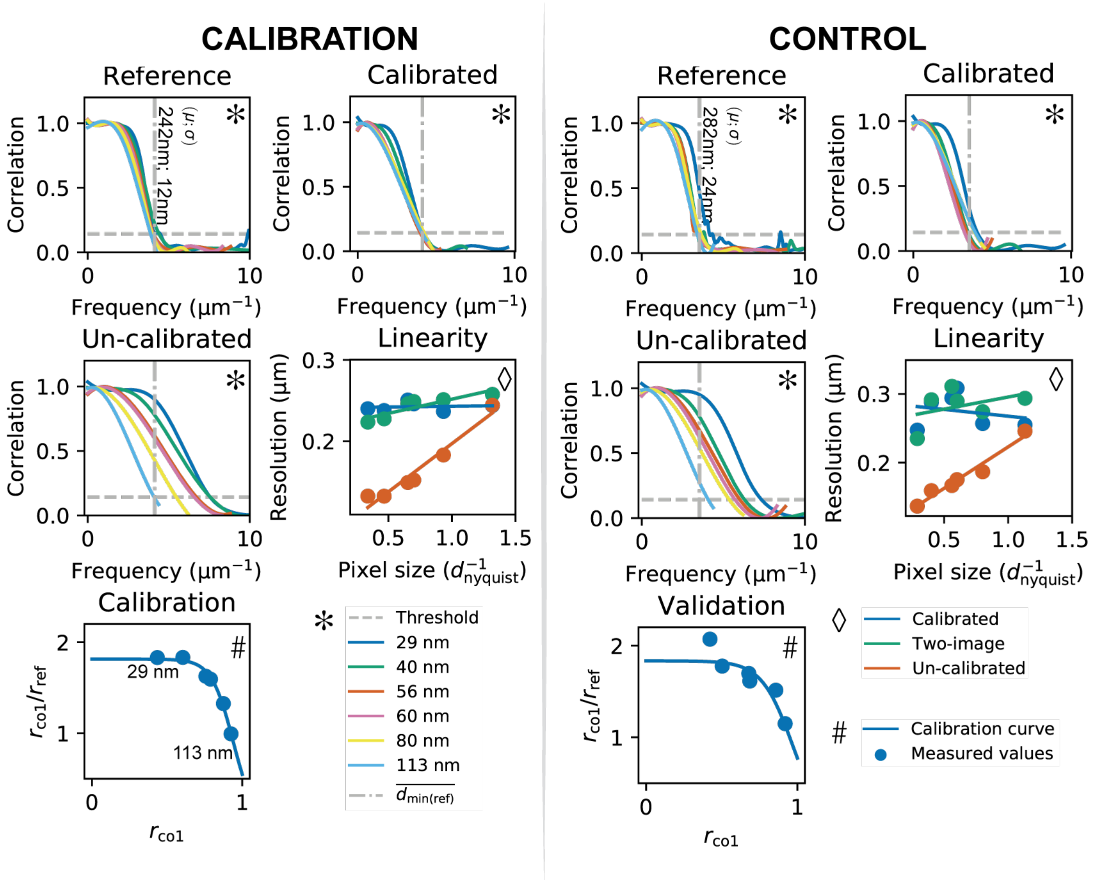

Supplementary Figure 3. *Calibrating one-image FRC.* The one-image FRC was calibrated with confocal image datasets that contain images from a fixed field-of-view and focal plane (constant resolution), but with several pixel sizes. Such images allowed us to obtain reference points for the calculation of a calibration curve, which relates each resolution value obtained with one-image FRC ($d_{\min(\mathrm{co1})}$, Un-calibrated), to a correct reference value ($d_{\min(\mathrm{ref})}$, Reference). For the calibration, first a scatter of $\overline{d_{\min(\mathrm{ref})}}/d_{\min(\mathrm{co1})}$ was plotted as a function of the one-image FRC cut-off frequency ($r_{\min(\mathrm{co1})}$), after which a curve $f(r_{\min(\mathrm{co1})}) = (ae^{cr_{\min(\mathrm{co1})}-b} + d)^{-1}$ was fit to the data (a=0.96, b=0.98, c=13.9, d=0.55); $\overline{d_{\min(\mathrm{ref})}}$ is the average resolution measured with two-image FRC. In the figure, the resolution ratio is expressed on a normalized frequency scale ($r_{\mathrm{co1}}/r_{\mathrm{ref}}||r_{\mathrm{co1}}; r_{\mathrm{ref}} \in [0,1]$) – please see Supplementary Note 2 for details. The calibration curve was obtained with confocal images of a cell with intermediate filaments stained, ($d_{\min(\mathrm{ref})}$= 242 nm; 12 nm ($\mu;\sigma$) (CALIBRATION). The Calibrated one-image FRC shows good accuracy and linearity, as shown in the Linearity plot ($d_{\mathrm{nyquist}} = \overline{d_{\min(\mathrm{ref})}}/2\sqrt{2}$). A control experiment was then performed, by applying the same calibration curve to a second set of images, of a cell with microtubules stained ($d_{\min(\mathrm{ref})}$ = 282 nm; 24 nm ($\mu;\sigma$)) (CONTROL). The measures in the second image series are rather noisy, as there are some saturation effects visible in the two-image FRC curves [1], but the calibration is accurate.

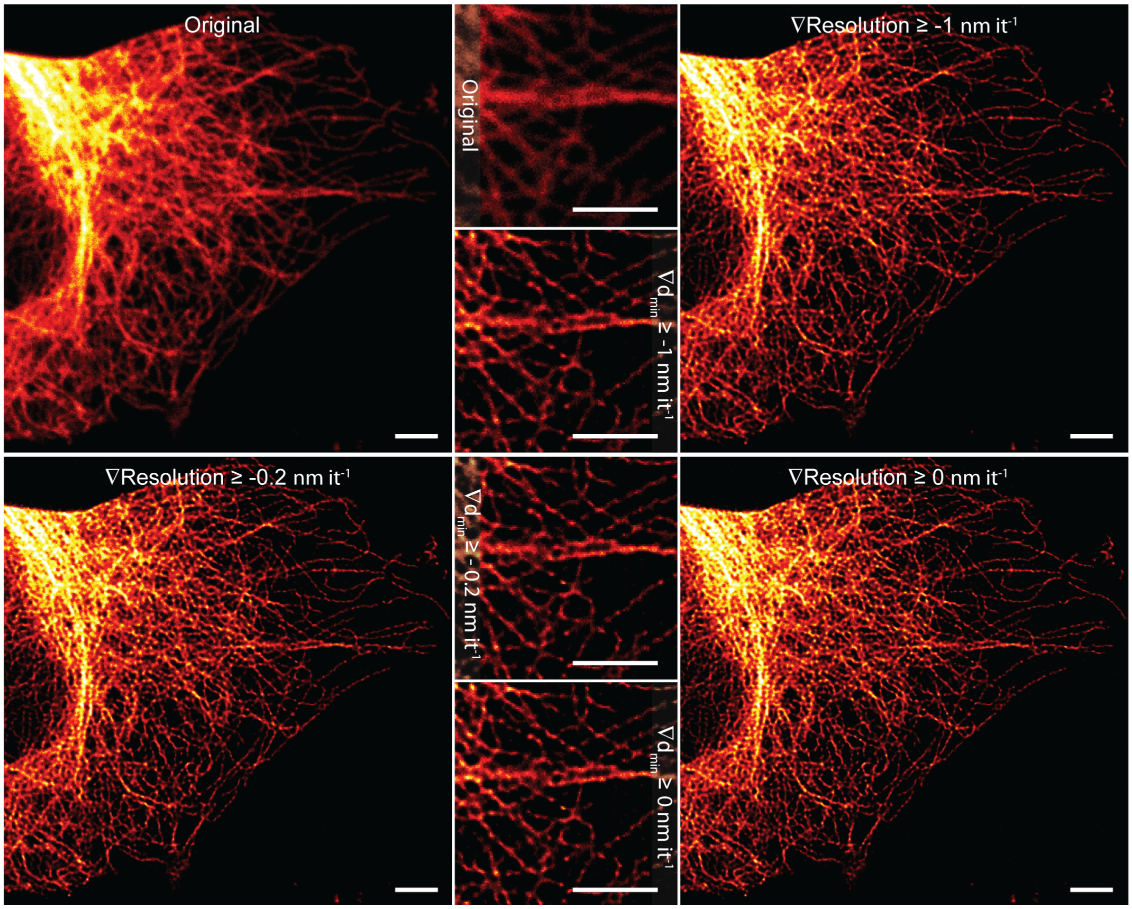

Supplementary Figure 4. *Comparing deconvolution results with different stopping conditions.* A microtubules stained fixed cell image was deconvolved with the blind RL algorithm (fixed PSF). Results at three alternative stopping positions are compared. The three different ∇Resolution (i.e. ∇d_{min}) values are reached after 34, 58 and 92 iterations, respectively. Only slight improvement of image quality after the first threshold. Scale bars 3 μm.

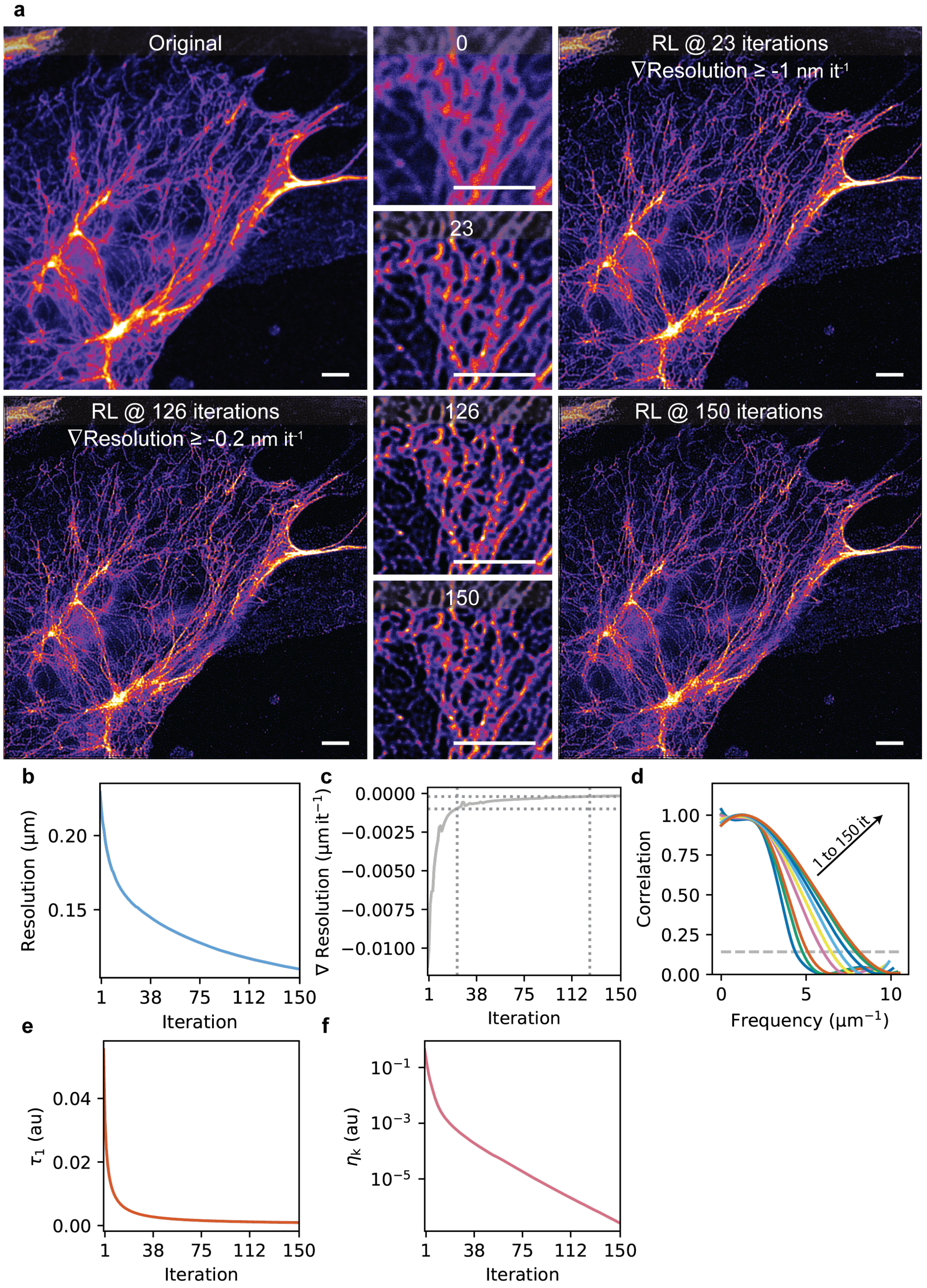

Supplementary Figure 5. *Observing the progress of blind RL deconvolution in a confocal image of a vimentin stained fixed cell.* An image of a fixed vimentin stained cell (29 nm pixel size) is deconvolved with blind RL deconvolution (PSF_{FWHM} = 240 nm). In a) deconvolution results after 0 (original image), 23, 126 and 150 deconvolution iterations are compared. In b) the FRC resolution value is plotted as a function of iteration, and in c) the first derivative of b) is shown. The dashed vertical lines in c) denote iterations (23, 126) that correspond to points at which the ∇Resolution value reaches the -1 nm it^{-1} and -0.2 nm it^{-1} thresholds. In d) FRC curves are shown for a subset of the deconvolution results. In e) τ_1 and f) η_k curves are shown. Scale bars 3 μm.

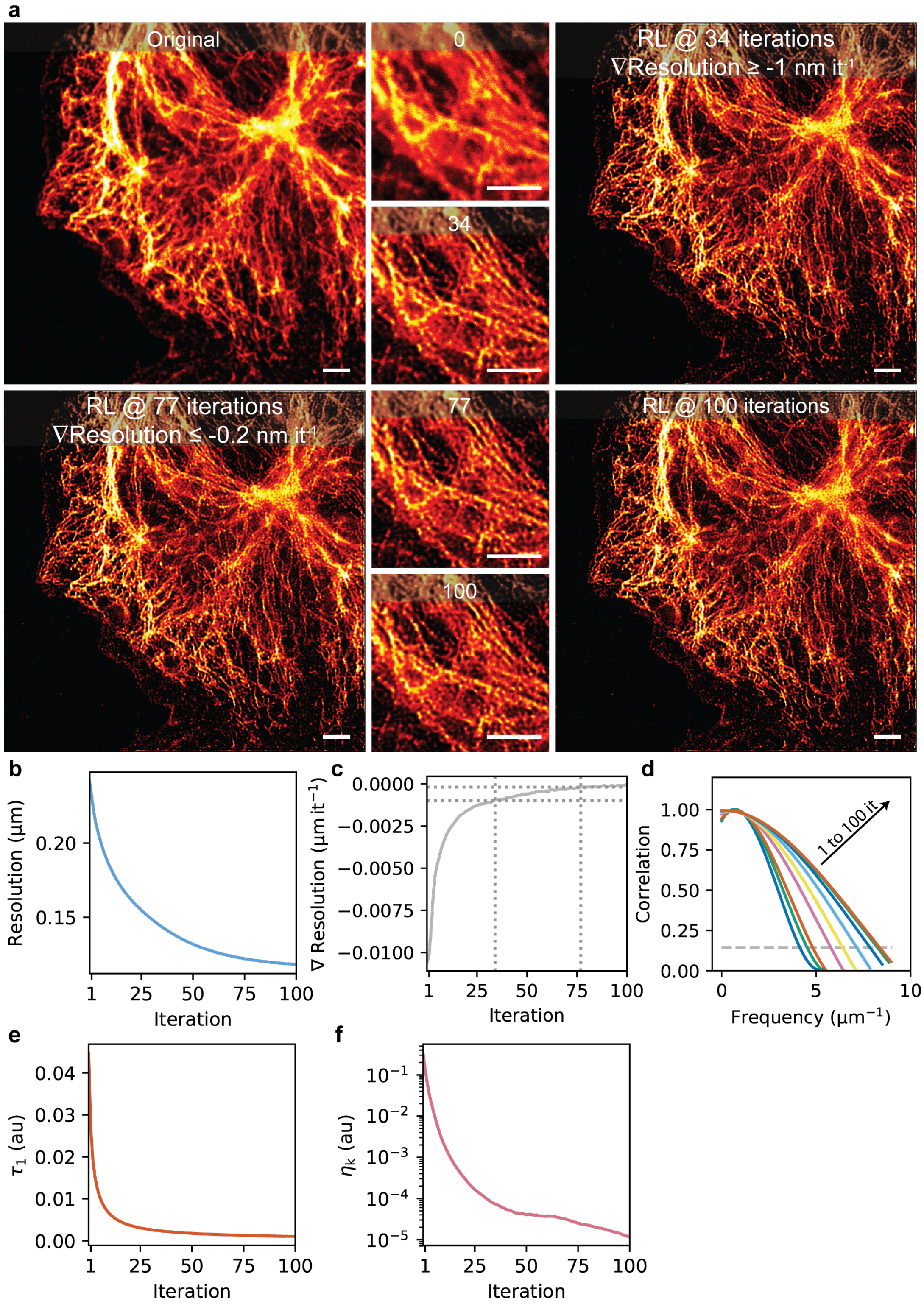

Supplementary Figure 6. *Observing the progress of blind RL deconvolution in a widefield image of a vimentin stained fixed cell.* A widefield image of a fixed vimentin stained cell (56 nm pixel size) is deconvolved with blind RL deconvolution (PSF_{FWHM} = 250 nm). In a) deconvolution results after 0 (original image), 34, 77 and 100 deconvolution iterations are compared. In b) the FRC resolution value is plotted as a function of iteration, and in c) the first derivative of b) is shown. The dashed vertical lines in b,c) denote iterations (34, 77), that correspond to points at which the ∇Resolution value reaches the -1 nm it^{-1} and -0.2 nm it^{-1} thresholds. In d) FRC curves are shown for a subset of the deconvolution results. In e) τ_1 and f) η_k curves are shown. Scale bars 3 μm.

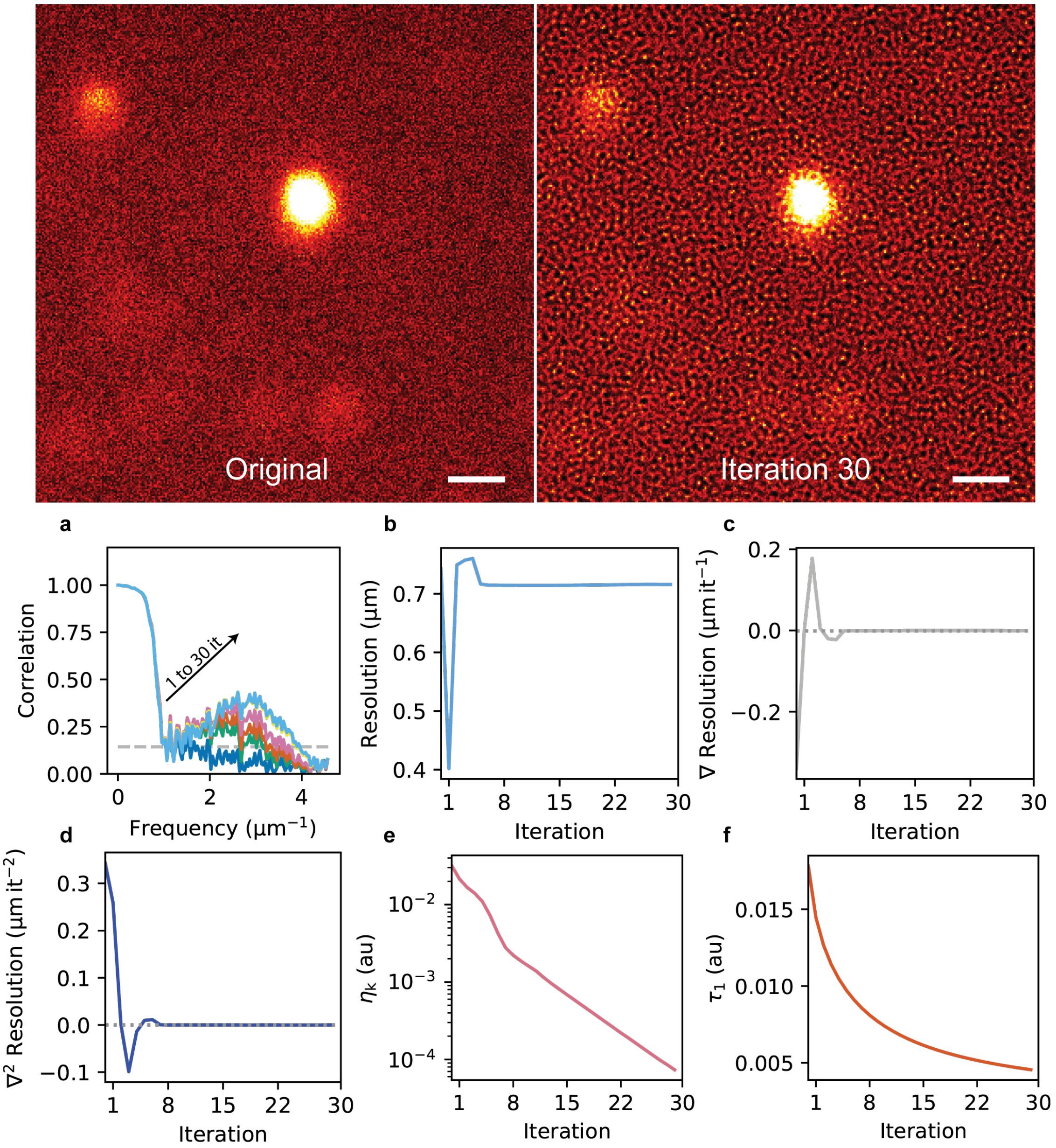

Supplementary Figure 7. *Simulating the effect of noise background in RL deconvolution.* The effect of background noise to FRC measures was simulated by running RL deconvolution with a small PSF (FWHM = 230 nm) on an image of a uniform fluorescence layer. Such an image is more or less a worst kind of an input for a RL algorithm, as there is no useful signal to fit and thus the algorithm starts to immediately fit the noise. The FRC measures a) show a noise artefact starting to form, right after the first iteration. The FRC curves are shown without any smoothing, to highlight that all the curves fall below the 1/7 threshold between the two peaks. The FRC resolution measures of the intermediate deconvolution estimates b) shows that the deconvolution reaches maximum resolution after one iteration. The first c) and second d) derivatives of b) both correctly indicate that the deconvolution should stop. η_k e) and τ_1 f) do not work. Scale bars 3 μm.

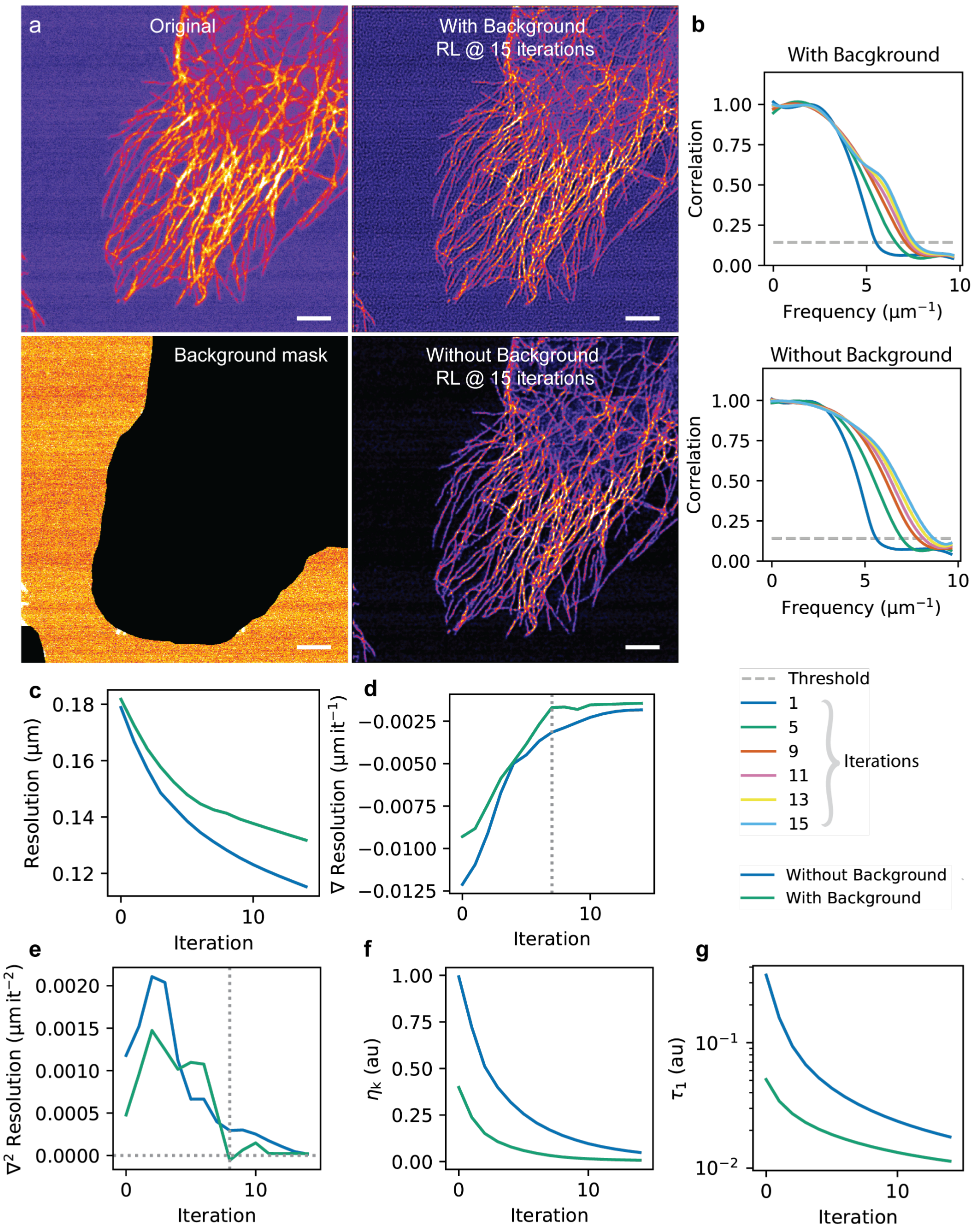

Supplementary Figure 8. *RL background correction and FRC.* RL deconvolution with and without background correction is run on a noisy HeLa cell image with microtubulin staining. Two-image FRC was used to evaluate the deconvolution progress in this example, because we wanted to access the entire frequency range. The deconvolution was run separately with the two images and then the FRC was calculated afterwards, for each intermediate result image pair. For the background correction, the empty areas of an image are first separated from those containing details with a spatial mask, after which the background is estimated as the average signal value in the empty area. The spatial masking technique is explained in detail in [2]. As shown in a) the background correction has a dramatic effect on the deconvolution quality. The FRC measures of the intermediate deconvolution results shown in b), reveal a clear background noise artefact that starts to form after 7 iterations; the background correction effectively removes it. FRC resolution measures on the intermediate deconvolution estimates in c) show that RL with background correction reaches a much higher resolution. The first derivative of c), shown in d), shows a clear discontinuity at 7 iterations in the RL deconvolution without background estimation (With Background), which is indicative of the appearance of the noise bump. The second derivative of c), shown in e), reveals the edge as a zero crossing. Neither η_k f) or τ_1 g) give similar qualitative information. Scale bars 3 μm.

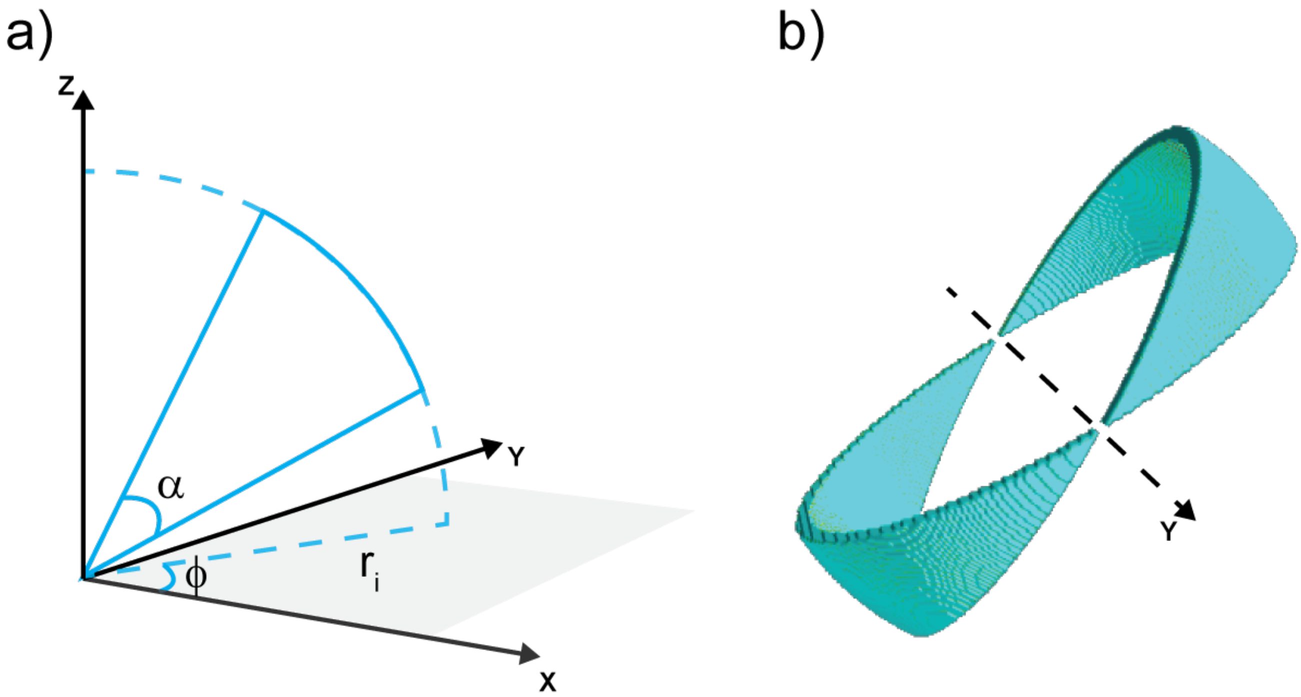

Supplementary Figure 9. *Illustrating the principle of SFSC Fourier Shell Indexing.* In a) the indexing parameters in 3D coordinate system are explained. α (elevation), defines the thickness of the section and the orientation of the shell in relation to XY plane; the orientation is a multiple of α and is changed as a part of the iteration process. ϕ (azimuth) defines the rotation axis on the XY plane. r_{i} is the radius of the ith shell. In b) volume rendering of a single indexing structure is shown; the rotation in this case is done around the Y axis.

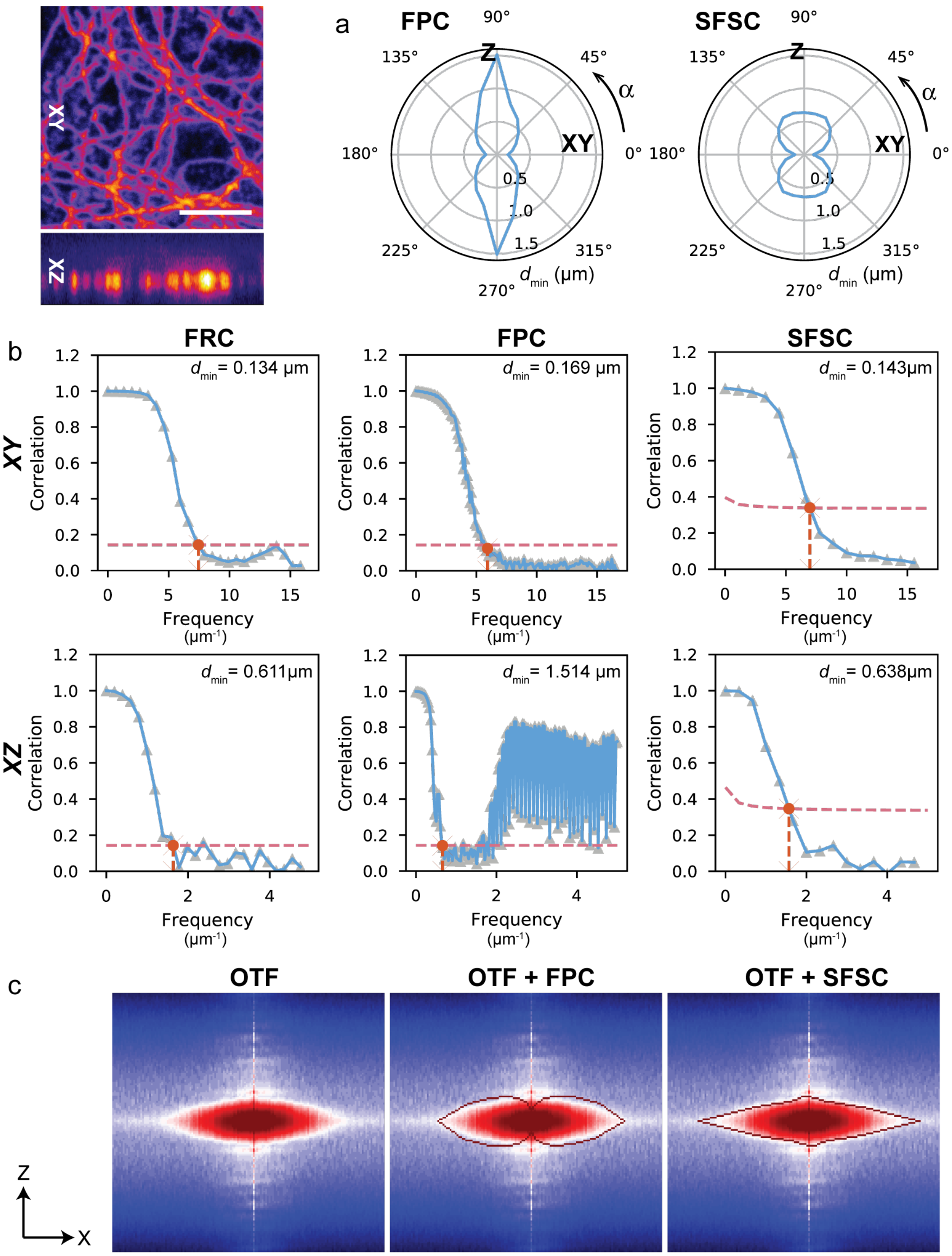

Supplementary Figure 10. *3D resolution measurements with FRC, SFSC and FPC.* The resolution measures with FRC, but FPC and SFSC are compared on the STED image stack. In a) 3D resolution measures produced by FPC and SFSC are shown in a polar plot. Both methods correctly reveal the resolution anisotropy, FPC appears to underestimate the resolution in the axial direction. In b) FRC, FPC, and SFSC measures are compared at XY and XZ orientations. FRC and SFSC are in good agreement, but FPC produces a somewhat pessimistic numeric value in XZ. In c) the FPC and SFSC results are plotted as contours on top of the maximum projection (along y-axis) of the Fourier transform of the STED image. The maximum projection approximates the optical transfer function (OTF) of the microscope. SFSC faithfully retraces the white outline of the OTF, whereas FPC is not quite as accurate. Strong artefacts are visible in the axial (z) direction of the OTF, which explains the noise in the FPC curve in b). Scale bar 3 μm.

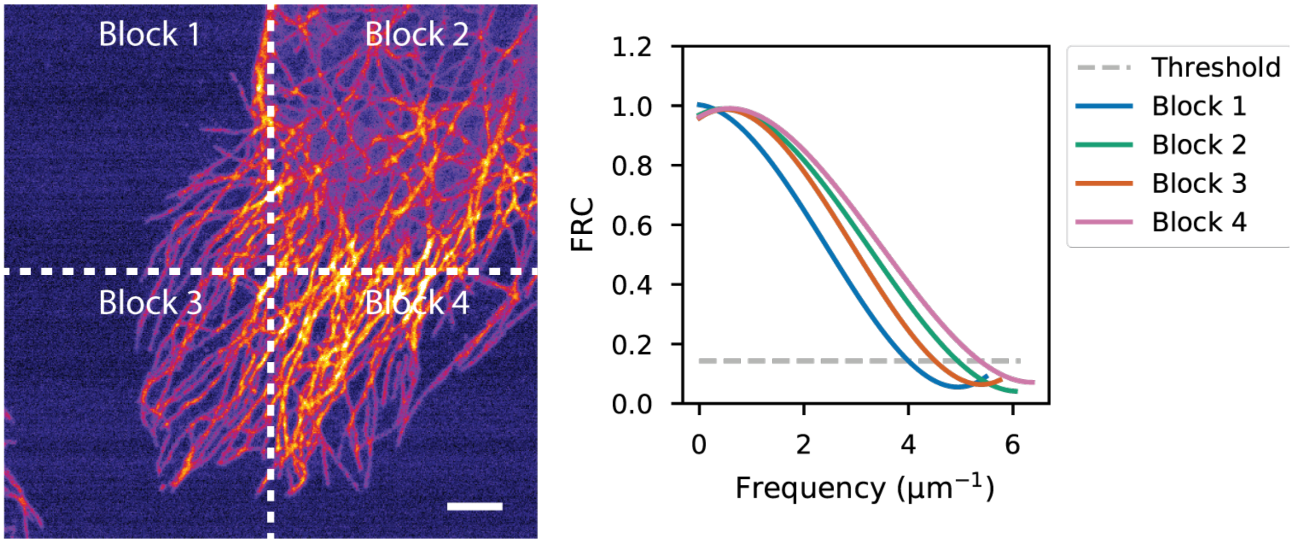

Supplementary Figure 11. *Block based FRC*. It is demonstrated with the microtubulin stained HeLa cell that it is possible to easily calculate local resolution values with the (one-image) FRC. Scale bar 3 μm.

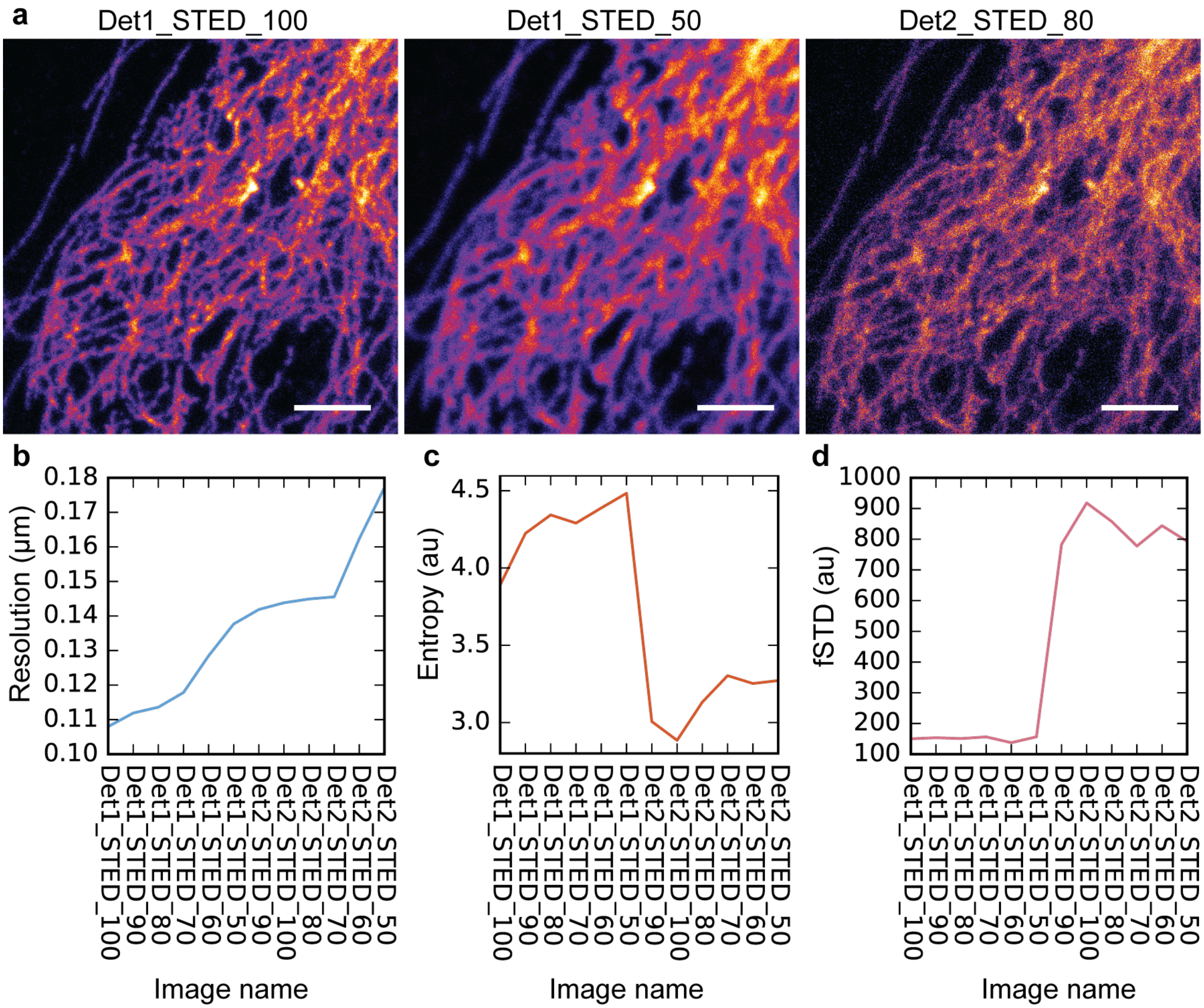

Supplementary Figure 12. *FRC can help in quantitative image quality assessment.* The FRC measurement a) is compared against two image quality related parameters – sptial domain entropy b) and fSTD c) [2] – on a dataset containing STED super-resolution images with two different detector configurations and five different depletion beam intensities. The spatial domain entropy measure is very sensitive to contrast changes, whereas the fSTD measure reacts strongly to noise and blur. The information given by the three measures is very complementary; one might e.g. select a ”good” image in the series, based on some sort of combination of these three parameters. Scale bars 3 μm.

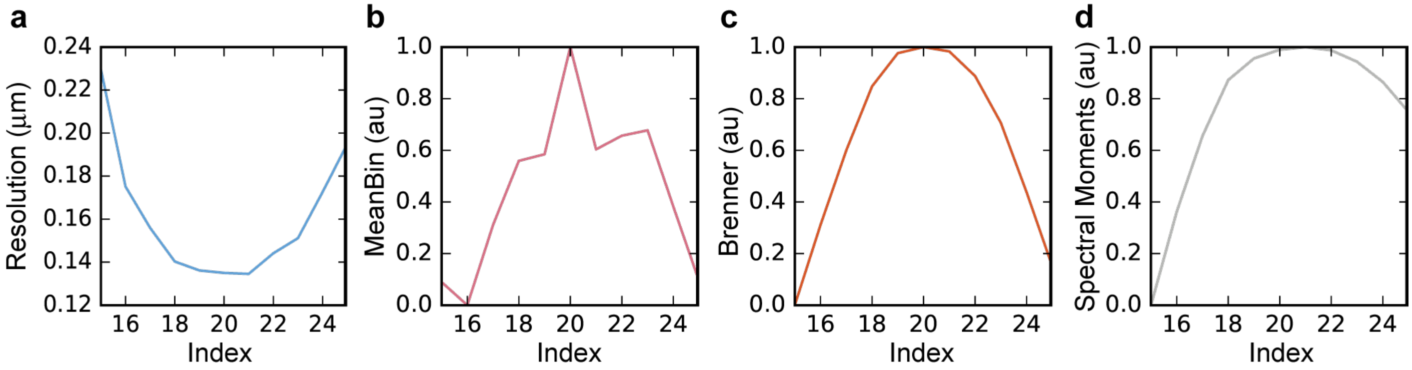

Supplementary Figure 13. *FRC dependency on focal position in a 3D stack* The resolution measured with FRC at different axial positions in the STED image stack (main article Figure 5a), is shown a) and compared to several auto-focus metrics: Meanbin b) [2], Brenner c) [3] and spectral moments d) [4].

Supplementary Note 1. The effect of image shift in one-image FRC/FSC

The image splitting method that is used for one-image FRC produces a single pixel shift both in x and y directions. Here we try to understand what such shift does to the FRC/FSC curve. According to the Fourier shift theorem, a shift of (y_0, x_0) pixels of image f_i in the spatial domain, will result into $e^{-i2\pi sr/N}$ frequency dependent phase modulation of its Fourier transform. r is equivalent to the radius in the FRC/FSC equation; it should actually be expressed as a vector, but here we abuse the notation a little bit as the summation over rings/spheres in FRC/FSC calculation essentially reduces the dimensionality of a signal to 1D. $s = \sqrt{x_0^2 + y_0^2}$ equivalently is the total length of the shift. The FRC/FSC equation (main article Eq. 1) can be modified to include the shift as follows:

$$\text{FRC/FSC}_{12}(r_\mathrm{i}) = \frac{\sum_{r \in r_\mathrm{i}} F_1(r) \cdot (F_2(r) \cdot e^{-i2\pi sr/N})^*}{\sqrt{\sum_{r \in r_\mathrm{i}} F_1^2(r) \cdot \sum_{r \in r_\mathrm{i}} (F_2 \cdot e^{-i2\pi sr/N})^2(r)}} \tag{1}$$

Because $(e^{-i2\pi sr/N})^2 = 1$, it can be altogether ignored in the denominator. On the other hand, because both F_1 and F_2 are Friedel symmetric, the result of summation over Fourier ring/sphere will always be real. The numerator can thus be rewritten as

$$\sum_{r \in r_i} Re\left\{(F_1 \cdot F_2^*)(r) \cdot e^{-i2\pi sr/N}\right\} \tag{2}$$

Let's now make two variable changes $F_\mathrm{cc} = F_1 \cdot F_2^*$ and $D = N/2$; substitution to D is motivated by the fact that $0 \le r_\mathrm{i} < N/2$. The numerator can now written to form

$$\sum_{r \in r_\mathrm{i}} Re\left\{F_{cc}(r) \cdot e^{-i\pi r/(D/s)}\right\} \tag{3}$$

, which ends up being a discrete cosine transform (DCT-I), of length D/s. It is known that DCT-I of length D is exactly equivalent to a DFT of length $2D-2$ with even symmetry. The shift s adds a modulation to the DCT that essentially makes the frequency step size larger, thus compressing the spectrum.

Supplementary Note 2. Calibration of one-image FRC to a known reference

In order to enable reasonably accurate quantitative measures with one-image FRC, it needs to be calibrated. Here we discuss the basic theory behind the calibration process.

The maximum frequency in an image, allowed by a given sampling density can be described by

$$f_{\text{max}} = \frac{1}{2d_{\text{px}}} \tag{4}$$

, where d_{px} is the pixel size in an image. This does not mean that the image would necessarily have such frequencies, if for example the sampling density is much higher than required by the image resolution, i.e. $f_{\text{nyquist}} = 2\sqrt{2}/d_{\text{min}}$, where d_{min} denotes the image resolution.

Any frequency f in an image can be described as a fraction of the maximum frequency

$$r = \frac{f}{f_{\text{max}}} \tag{5}$$

, and thus one obtains a normalized frequency scale $r \in [0, 1]$. In the context of FRC, r can be considered as the radius of the given Fourier ring. Such normalized representation is comfortable, because it is independent of image dimensions or the physical scale (pixel size).

The image resolution can now be written in terms of the radius r and the maximum frequency resolution d_{min} one can write f_{max}

$$d_{\text{min}} = \frac{1}{f_{\text{co}}} = \frac{1}{r_{\text{co}} f_{\text{max}}} = \frac{2d_{\text{px}}}{r_{\text{co}}} \tag{6}$$

, where f_{co} and r_{co} denote the cut-off frequency, in physical and normalized scales.

Now, in order to obtain a calibration for the one-image FRC, one needs to find out, how the measured cut-off frequency value differs from a correct reference value. We describe the relationship in terms of the radii

$$f(r_{\text{co1}}) = \frac{r_{\text{co1}}}{r_{\text{ref}}} \tag{7}$$

, where r_{co1} is the cut-off value obtained with one-image FRC, r_{ref} is the reference value and $f(r_{\text{co}})$ is the calibration curve. In order to obtain an estimate for the calibration curve, one needs a number of paired values of r_{co} and r_{ref}; the calibration can then be extended from the reference points to the whole frequency band, by curve fitting.

As expressed in (Eq. 6), one can change the values of r_{co1} and r_{ref}, either by changing the image resolution, or the pixel size. In a confocal microscope the image resolution is determined mainly by the fluorescence wavelength and the numerical aperture of the microscope objective – and it is limited by diffraction to $\sim d_{min} = \lambda/2\text{NA}$, where λ is the wavelength of light and NA the numerical aperture. In a fixed field-of-view and focal position (defocus will decrease resolution), assuming that no changes are made to the optical configuration, the resolution is thus nearly constant. The pixel size on the other hand, is a parameter that one can adjust freely. Thus, by keeping the field-of-view and focal position fixed, and varying the pixel size, it is possible to obtain a set of reference measurements that can be used for the calibration. Finally, the resolution Eq. 6 can be reformatted to include the calibration curve

$$d_{\text{min}} = \frac{2d_{\text{px}} f(r_{\text{co1}})}{r_{\text{co1}}} \tag{8}$$

As a further note, instead of using the normalized radii, Eq. 7 can be expressed in physical scale, either in terms of distances (spatial domain) or frequencies (frequency):

$$f(r_{co}) = \frac{f_{\mathrm{co1}}}{f_{\mathrm{ref}}} = \frac{d_{\mathrm{min(ref)}}}{d_{\mathrm{min(co1)}}} \tag{9}$$

In our calibration, we used the average resolution value of two-image FRC $\overline{d_{\mathrm{min(ref)}}}$, as the reference value. Therefore it was easier to express the ratio in terms of spatial units. The calibration curve however is unitless, and does not depend on the spatial scale.

Supplementary Note 3. Test image descriptions

The test images consist of various types of confocal as well as STED microscope images that were acquired with a variety of commercial and custom-built microscopes. A short descriptions of the samples and the image acquisition parameters are given below:

- A fixed HeLa cell with alpha-tubulin stained, using Star488 secondary antibodies (Abberior, Germany). Sample was imaged with a Nikon A1 confocal microscope, with excitation wavelength 488nm, 60x/1.4 oil immersion objective, 0.4 AU pinhole and GaAsP detector.The image size is 512x512 and pixel size 51x51 nm. This image was used in Figure 1, Supplementary Figures 2, 8 & 11.

  A sample with tubulin cytoskeleton stained with Star-635P (Abberior, Germany). The sample was imaged with Abberior Instruments Expert Line STED system, excitation wavelength 633 nm, depletion wavelength 775 nm, 100x/1.4 (UPLSAPO100XO) objective. The image size (x,y,z) is 1024x972x30 and voxel size 30x30x100 nm. This image was used in Fig 4a, Supplementary Figures 10 & 13.
- A sample of pollen, imaged with a Nikon A1 confocal microscope, with excitation wavelength 488 nm, 40x/1.2 water immersion objective and GaAsP detector. Due to custom intermediate optics on the illumination/detection path, the objective was underfilled, to produce an effective NA of approximately 0.5-0.6. Image size is 512x512x181 and voxel size 78x78x250 nm (x,y,z). This image was used in Figure 4b.
- A fixed HeLa cell with tubulin cytoskeleton stained with Star635. The sample was imaged with Leica TCS SP5 STED, 100x/1.4 oil immersion objective (HCX PL APO CS), pinhole 1.0 AU, excitation wavelength 635 nm. The depletion laser wavelength was set to 765 nm and its intensity was varied between 50-100% – the same field of view was imaged sequentially with two different detectors. The image size is 700x700 and pixel size 15x15 nm. These images were used in Supplementary Figure 12.
- A fixed cell with intermediate filaments (vimentin) cytoskeleton stained with Star635. The sample was imaged with Abberior Instruments Expert Line STED system, excitation wavelength 633 nm, 100x/1.4 (UPLSAPO100XO) objective. The dataset used in Figure 2 and Supplementary Figures 3 & 5 consists of images with different pixel sizes (29 → 113 nm; dimensions 1389x1389 → 354x354). The widefield image in Supplementary Figure 6 was acquired with 56 nm pixel size, and the optical pinhole was opened to remove the optical sectioning effect. These images were used in Figure 2, Supplementary Figures 3, 5 & 6.
- A fixed cell with microtubulin cytoskeleton stained with Star635. The sample was imaged with Abberior Instruments Expert Line STED system, excitation wavelength 633 nm, 100x/1.4 (UPLSAPO100XO) objective. The dataset consists of images with different pixel sizes (29 → 113 nm; dimensions 1389x1389 → 354x354). These images were used in Figure 3, Supplementary Figure 3
- A Atto647N fluorescent layer imaged with Abberior Instruments Expert Line STED system, excitation wavelength 633nm, 100x/1.4 (UPLSAPO100XO) objective. The image size is 500x500 and pixel size 60x60nm. This image was used in Supplementary Figure 7.

Supplementary Note 4. MIPLIB open-source image analysis software

MIPLIB is an evolution from the *SuperTomo* software [5] that was originally written for the specific purpose of performing multi-view tomographic reconstructions with large 3D STED microscopy datasets. Over the years its scope and features have slowly expanded towards a general purpose microscopy image analysis and processing library. In addition to the new FRC/FSC features, we recently decided to consolidate several previously separate image analysis and processing packages, dealing with e.g. quantitative image quality analysis [2] , correlative microscopy [6] and image deconvolution into MIPLIB as well.

The library is being made available under FreeBSD open source license, and it can be downloaded at: https://github.com/sakoho81/miplib

Regarding iteration in polar/spherical coordinate space. In order to conveniently calculate the (S)FSC measures, flexible methods to index the 3D Fourier space needed to be created. To this end we created a series of spherical-coordinate-system-based Fourier Shell iterators in the MIPLIB software. The iterators produce the sequence of 3D indexing structures that are needed to extract voxels on a given shell/section from the Fourier space image for FSC calculation. The iterators were designed to be interchangeable – any one of them can be used in the FSC implementation to produce the desired behaviour. In addition to the regular FSC and SFSC iterators, we also implemented special ones to exclude pixels in certain orientations – this is necessary as microscope images often contain artefacts generated e.g. by the mechanical movement of the xyz - scanning apparatus; the artefact become visible in the FRC/FSC measures as higher than normal resolution values. Excluding pixels in the direction of the optical axis seems to be especially important, as the axial scanning steps (piezo) as well as possible interpolation artefacts otherwise compromise the resolution measure. With each iterator the width of the sections, the thickness of the shells (bin size), as well as the width of the possible exclusion area can be freely selected.

Similar interchangeable iterator scheme is used in the FRC implementation as well, the main difference is that the iterators work in polar coordinate system, and only one cross-correlation curve is calculated for every image. It may in some instances be of interest to exclude certain parts of the Fourier rings as well, if a particular orientation of features is of interest, or if there are artefacts in a certain direction affecting the FRC results.

Regarding FRC/FSC analysis. The FRC/FSC analysis is a multi-stage process, in which first the FRC/FSC histograms are generated, after which the they are analyzed to find out the resolution values. The FRC/FSC datasets consist of the cross-correlation histogram and a list of the number of pixels/voxels on each ring/shell; the latter information is needed to calculate the threshold curves. The FRC/FSC analysis workflow is described algorithmically in Algorithm 1.

Algorithm 1 Simple pseudocode for our FRC/FSC analysis algorithm. Words in italics denote variable names.

```
for each Orientation in Iterator.Orientations do
    calculate FRC/FSC Dataset
    save to DataCollection
end for
for each Dataset in DataCollection do
    fit a curve to Dataset.Correlation
    calculate Threshold from Dataset.NPoints
    fit curve to Threshold
    find an intersection of the two curves
end for
```

The curve fitting has been implemented in two different ways: (I) a standard linear model fitting, with an polynomial function of the order of n and (II) a piece-wise (smoothed) splines based fitting method. The former

method makes it possible to enforce a given shape for the resolution curve , whereas the latter is more robust, especially with very noisy data, into which it is hard to reliably fit a polynomial without some sort of pre-filtering or cropping. We almost exclusively use the smoothed splines as they produce nice looking FRC/FSC curves almost with any kind of data.

The intersection of the cross-correlation and the threshold curves is calculated by minimizing $|\text{FRC/FSC}(x) - \text{Th}(x)|$, where FRC/FSC(x) and Th(x) are the values of the correlation curve and the threshold curve at frequency x, respectively. The optimization is done with a classical Simplex algorithm [7]. In order to reduce the number of iterations, the optimization is started at a frequency x at which the FRC/FSC correlation value in the original histogram is just above the threshold curve. MIPLIB supports fixed (1/7 or any other lever), $n\sigma$ based as well as SNR_e based resolution threshold equations [8].

The test images consist of various types of confocal as well as STED microscope images that were acquired with a variety of commercial and custom-built microscopes. A short description of the samples and the image acquisition parameters is given below:

References

[1] Tortarolo, G., Castello, M., Diaspro, A., Koho, S. & Vicidomini, G. Evaluating image resolution in stimulated emission depletion microscopy. *Optica, OPTICA* **5**, 32–35 (2018).

[2] Koho, S., Fazeli, E., Eriksson, J. E. & Hänninen, P. E. Image quality ranking method for microscopy. *Sci. Rep.* **6**, 28962 (2016).

[3] Brenner, J. F. *et al.* An automated microscope for cytologic research a preliminary evaluation. *J. Histochem. Cytochem.* **24**, 100–111 (1976).

[4] Firestone, L., Cook, K., Culp, K., Talsania, N. & Preston, K., Jr. Comparison of autofocus methods for automated microscopy. *Cytometry* **12**, 195–206 (1991).

[5] Koho, S., Deguchi, T. & Hänninen, P. E. A software tool for tomographic axial superresolution in STED microscopy. *J. Microsc.* **260**, 208–218 (2015).

[6] Prabhakar, N. *et al.* STED-TEM correlative microscopy leveraging nanodiamonds as intracellular Dual-Contrast markers. *Small* **14**, 1701807 (2018).

[7] Nelder, J. A. & Mead, R. A simplex method for function minimization. *Comput. J.* **7**, 308–313 (1965).

[8] van Heel, M. & Schatz, M. Fourier shell correlation threshold criteria. *J. Struct. Biol.* **151**, 250–262 (2005).
